# Intertester reliability of a movement impairment-based classification system for individuals with shoulder pain

**DOI:** 10.1142/S1013702520500067

**Published:** 2020-01-28

**Authors:** Patitta Torwichien, Mantana Vongsirinavarat, Prasert Sakulsriprasert, Sirikarn Somprasong

**Affiliations:** 1 Faculty of Physical Therapy, Mahidol University, Nakhon Pathom 73170, Thailand; mantana.von@mahidol.edu

**Keywords:** Movement system impairment, reliability, shoulder pain classification, subacromial impingement syndrome

## Abstract

**Background:**

Other than pathoanatomical diagnosis, physical therapy managements need the diagnosis of movement-related impairments for guiding treatment interventions. The classification system of the Movement System Impairment (MSI) has been adopted to label the musculoskeletal disorders in physical therapy practice. However, reliability study of this classification system in individuals with shoulder pain has not been reported in the literature.

**Objective:**

This paper investigated the intertester reliability of the diagnosis based on the MSI classification system in individuals with shoulder pain.

**Methods:**

The patients with shoulder pain, between the ages 18–60 years, were recruited if he or she had pain between 30 and 70 on the 100 mm visual analog scale for at least three months. The examiners who were two physical therapists with different clinical experiences received a standardized training program. They independently examined 45 patients in random order. Each patient was examined by both therapists on the same day. The standardized examination scheme based on the MSI approach was used. Patients were identified to subgroup syndromes according to scapular and humeral syndromes and also determining their subcategory syndromes. Six scapular subcategory syndromes included downward rotated, depressed, abducted, wing, internal rotated/anterior tilted, and elevated. Three humeral subcategory syndromes were anterior glide, superior glide, and medial rotated. More than one subgroup and subcategory of syndromes could be identified in each patient. The test results of each session were blinded to another therapist. The percentages of agreement and kappa statistic were determined.

**Results:**

The results showed that agreement levels in identifying subgroup syndromes was fair (71.11% agreement, kappa coefficient = 0.34) and classifying subcategories syndromes were poor to substantial (73.33–91.11% agreement, kappa coefficient = 0.20–0.66). The overall agreement and kappa value of the MSI classification of subcategory syndromes was poor (kappa coefficient = 0.11; 95% CI 0.05–0.18). The agreement level of subcategories for scapular depression and humeral superior glide syndromes was substantial. The scapular winging, depression, and downward rotation were the three syndromes that were most frequently identified by both the examiners.

**Conclusion:**

The intertester reliability between therapists with different experience according to the MSI approach for shoulder pain classification was generally acceptable to poor due to the nature of the classification system. The standardized procedure and intensive training can be used for inculcating novice therapists with adequate level of intertester reliability of examination.

## Introduction

Shoulder pain is one of the most common and is ranked third among musculoskeletal complaints after back and neck pain.^1,2^ Clinically, shoulder pain is varied in terms of symptom behaviors^3^ and their pathoanatomical as well as the pathokinesiological lesions.^4,5^ Evidences showed various alterations of movements during arm raising and lowering in patients with shoulder pain especially subacromial impingement syndrome (SIS).^6,7^ Nowadays, the shoulder rehabilitation program focuses not only on treating the structures causing pain but correcting the abnormal alignments and movements leading to the injury.

The concept of the Movement System Impairment (MSI) has been proposed by Sahrmann^8^ since 1980s and has been adopted among physical therapists. This approach is based on the kinesiopathological model which focuses on the identification of repetitive movements and sustained positions which are the primary cause of tissue injuries rather than the affected anatomical structures. According to the model, the movement system is composed of musculoskeletal, nervous, cardiopulmonary and endocrine systems interacting to produce normal biomechanics. However, specific directions of the repeated joint movement and sustained alignments are the inducers of soft tissue adaptations by changing tissue stiffness and extensibility associated with the loss of movement precision. Depending on the personal characteristics which are the individual modifiers, these factors lead to directional susceptible to joint movements and cause movement impairments. The repeated low-magnitude stress to the soft tissues then leads to tissue microtrauma and eventually progresses to tissue macrotrauma or pathology and leads to limitation of function.

Utilizing this approach, the therapist plays an important role in redesigning movements and managing the contributing factors of deviated movement causing pain. The diagnosis according to the MSI classification system provides the guidance for physical therapy intervention especially patient education regarding posture and movements, and specific therapeutic exercises. There was a case report of the MSI approach in patient with SIS illustrated its clinical utility in this condition.^9^ The specific application of MSI classification system to the shoulder region is described by the characteristics of syndromes and sub-categories syndrome as shown in Appendix A.^8^

To apply a classification system in clinic, the psychometric properties are needed to be confirmed. The reliability studies of the MSI approach with various study procedures were available on low back pain^10-12^ and knee pain.^13-15^ The results showed good to excellent intertester reliability (kappa coefficients (K)=0.61–0.71) for low back pain and substantial agreement between raters (K=0.66–0.71) for knee pain. However, the reliability of using the MSI classification system to identify the movement impairments in patients with shoulder pain is still lacking. Therefore, the objective of this study was to investigate the intertester reliability of the diagnosis based on the MSI classification system in individuals with shoulder pain.

## Materials and Methods

### Study design

This study was an observational cross-sectional study investigating intertester reliability of the standardized MSI-based examination to classify individuals with shoulder pain.

### Patients

The consecutive patient with shoulder pain aged between 18 and 60 years were recruited by convenience sampling method from the out-patient orthopedic clinic in a university hospital and a physical therapy center. Eligible patients had shoulder pain for at least three months and pain during movement rated between 30 and 70 on the 100 mm visual analog scale. The exclusion criteria were shoulder symptoms referred from cervical region, signs of acute inflammation or severe pain that resulted in difficulty to move the upper extremity, chronic adhesive capsulitis, suspected rotator cuff tears, suspected glenoid labrum tear, history of shoulder or neck surgery, fractures of shoulder-linked bones, observable scoliosis and severe kyphosis, history of the neurological conditions affecting movement, received corticosteroid injection on the shoulder within previous 30 days, and elite professional athletes and high-level weight training.

There were 45 patients (11 males, 34 females) with chronic shoulder pain eligible and agreed to participate. Although five patients had shoulder pain in both arms, the examinations randomly selected one of the shoulders for assessment. Most of the participants were right-hand dominant (91.11%) but the shoulder pain was more on the left side (64.44%). The disability level was measured using Disability of the Arm, Shoulder, and Hand (DASH) questionnaire. The characteristics of participants are presented in Table 1.

**Table 1.** Characteristics of the participants (n=45).

Characteristics	Mean	SD	% (n)
Age (years)	39.49	10.98	
Gender distribution	—	—	
Male			24.44 (11)
Female			75.56 (34)
Body mass index in kg/m^2^	24.26	3.94	—
Side of pain	—	—	
Right			35.56 (16)
Left			64.44 (29)
Duration of shoulder pain (months)	6.82	4.62	—
VAS (after testing)	3.2	2.11	—
DASH total score (%)	21.33	14.83	—
DASH work score (%)	21.17	20.27	—

*Note*: DASH=Disability of the Arm, Shoulder, and Hand, VAS=Visual Analog Scale.

All participants signed an informed consent before the study. The research protocol was approved by the Mahidol University Central Institutional Review Board and Siriraj Hospital (MU-CIRB 2016/074.1905). The estimation of sample size required was performed with the following criteria: a two-tailed test at the level of significance of α=0.05; minimal kappa of clinical significance of 0.40; expected kappa between examiners of 0.80; and expected agreement between examiners of 0.70.^16^ The appropriate number of sample size for this study at 80% sufficient power was 48. However, three patients refused to participate in the study during data collection therefore total participants in this study were 45.

### The MSI standardized examination procedure

The MSI standardized examination of shoulder according to Sharmann’s textbook was used to classify patients with shoulder pain into specific categories of movement impairment syndromes.^8^ Two main parts of the examinations included (1) a series of alignment and movement tests in several positions to identify the possible movement impairments, and (2) tests of the strength and length of related muscles to identify the contributing factors. The operational definitions, procedures for each test item and the criteria for classifying the patients with the shoulder pain were delineated in a reference manual. The examination began with the primary test in which the patients were asked to perform preferred posture and movement pattern. The examiner observed the alignments and movements and the symptoms were notified. If the complaint was aggravated or increased during the primary test, the examiner immediately performed secondary test by correcting the alignment and/or movement. The movement was then observed and the symptoms were recorded again. The positive change of complaint during the secondary test would confirm that the alignment and/or movement pattern corrected was the possible cause of pain. The strength and length of muscles considered as the contributing factors of movement impairment were also tested. The repeated pattern of the test results indicated the movement impairment diagnosis. After completion of the examination, the patient was classified into subgroup and subcategory syndromes. The MSI classification system of shoulder consisted of two main subgroup syndromes i.e., scapular and humeral syndromes. Six scapular subcategory syndromes included downward rotated, depressed, abducted, wing, internal rotated/anterior tilted, and elevated. Three humeral subcategory syndromes were anterior glide, superior glide, and medial rotated.^8^ In this study, all possible syndromes and subcategory syndromes were identified in each patient and used for the reliability analysis.

### Examiners and training

Two registered physical therapists with different levels of experience in musculoskeletal field were the examiners in this study. The first examiner had 15 years of clinical experiences. She had taken a three day continuing education course on the shoulder MSI-based approach. The theory and practice of the MSI classification system as well as the categorized shoulder syndromes according to Sahrmann’s textbook was presented in the course. After attending the course, she had applied the approach in her clinical practice in the past three years. The second examiner had two years of clinical experience without formal education related to the MSI approach. Both examiners participated in a standardized training program which consisted of three main sessions; didactic (1 week), hands-on practice (4 weeks), and verification sessions (2 weeks). The didactic period aimed to review and clarify the related anatomy, biomechanics, the concept of MSI and the operational definition of each test item in the standardized examination. The hands-on practice session focused on using the MSI approach in both asymptomatic and symptomatic individuals and making decision of diagnosis according to the assigned criteria. Lastly, the verification session focused on diagnostic accuracy of classification verified by an expert instructor who had taken a continuing education course on the MSI-based shoulder classification as well as teaching and applying the MSI concept in her clinical practice for eight years. At the end of this session, both examiners were able to independently evaluate and classify all six symptomatic subjects for preliminary session, the agreement of their evaluations was perfect.

### Assessment procedure

All participants were screened to determine the eligibility by another physical therapist. The demographic data and clinical outcomes, including pain intensity and shoulder functions were interviewed and recorded. This information was not known by two examiners who performed the MSI examination procedure.

During the MSI assessment, the participants exposed their upper thoracic and shoulder regions i.e., females wearing sports bras and males taking off their shirts. The shoes were also taken off. Participants were asked to assume a natural relaxed standing on a reference line marking the feet position. Two examiners performed the same series of testing beginning with the alignment testing in standing position and used the adhesive markers to mark the superior and inferior angles of the scapula. These markers were completely removed after each examiner finished the examination.

Both examiners assessed each participant on the same day. The order of which examiner to perform first, the testing was randomly determined by drawing number from a sealed envelope. The first examiner evaluated the participants independently in a private room. After the first examiner finished the evaluation, the participants were asked to rest about 15 min and pain level was reevaluated by the therapist who performed the screening. Then, the second examiner evaluated the same participants independently in the same private room. The patients were emphasized not to mention any information about the previous testing session to the other examiner. After finished the examination, the participants were asked to determine their pain level again. All test results and final movement diagnoses were recorded in a standardized assessment form. Both examiners did not discuss about the evaluation procedures and were blinded to the results of each other during testing session. They determined the MIS classification both subgroup and subcategory syndromes based on the most consistent pattern of alignments and movements observed throughout the examination considering the symptom patterns of patients. The positive findings of syndromes and subcategory syndrome were the alignment and movement directions of the scapula and humerus that provoked symptom and reduced with the correction. The syndrome(s) of scapular and/or humeral were identified. Then, the specific subcategory of each syndrome was also specified according to the criteria (Appendix A). The MSI classification was not mutually exclusive, therefore more than one syndrome and subcategory syndrome could be identified in each patient.

### Data analysis

The statistical analysis was performed by using SPSS software (SPSS Inc. Release 2009. PASW Statistics for windows, Version 18.0, Chicago: SPSS Inc.). The descriptive statistics was used for determining the demographic data, shoulder functional activity and percentage of the frequency of the movement impairment in subgroups and their subcategories. The intertester reliability of the MSI classification system including subgroup and subcategory syndromes was calculated. The percent agreements of each subgroup and subcategory syndromes were reported and the kappa statistics was used as the chance-corrected agreement between examiners. The kappa values were interpreted as follows: less than 0.20 indicated slight agreements, 0.21–0.40 fair agreement, 0.41–0.60 moderate agreement, 0.61–0.80 substantial agreement, and more than 0.80 almost perfect agreement.^17^ The kappa value above 0.40 was generally considered acceptable.^12^ A p-value of<0.05 was considered statistically significant.

## Results

Both examiners identified all participants with shoulder pain as having impairments of both subgroup and subcategory syndromes of the MSI classification system. The percentage of agreement between both examiners of identifying the MSI syndromes was 100% in all participants. However, the number of participants without MSI was zero, therefore the kappa value was not computable.

To identify if the patients had scapular, humeral or both syndromes, the percent agreements was 71.11%, with fair level of chance correction agreement (K= 0.34, p= 0.003, 95% CI 0.00, 0.64). The frequencies and percentages of the MSI syndrome(s) identified by two examiners are presented in Table 2.

**Table 2.** Frequency and percentage of the MSI syndromes in patients with shoulder pain (n=45).

		Classification by Examiners 2		
	Subgroup syndromes	Scapular syndrome only	Humeral syndrome only	Both syndromes	Total
Classification by Examiner 1	Scapular syndrome only	26	0	8	34
		(57.78%)	(0%)	(17.78%)	(75.55%)
	Humeral syndrome only	0	0	0	0
	Both syndromes	3	2	6	11
		(6.67%)	(4.40%)	(13.30%)	(24.44%)
	Total	29	2	14	45
		(64.44%)	(4.44%)	(31.11%)	(100%)

In particulars, two examiners did not agree that patients had which syndrome in 13 of 45 shoulders. Examiner 1 identified scapular syndrome in all patients, 76% had scapular syndrome only and 24% had both syndromes. Examiner 2 identified scapular syndrome in 96% of patients, 64% scapular syndrome only, 31% had both syndromes, while 4% were determined as having humeral syndrome only.

For subcategory syndromes based on the MSI classification system, the frequencies and percentages of each MSI subcategory syndromes identified by two examiners are presented in Table 3.

**Table 3.** Frequency, percentage and the intertester reliability of the subcategory syndromes in patients with shoulder pain (n=45 shoulders).

	Examiners 1		Examiners 2		Intertester reliability	
Subcategory syndromes	n	%	n	%	% agreement	Kappa
Scapular downward rotation	15	33.33	18	40.00	80.00	0.57
Scapular depression	17	60.00	23	51.11	80.00	0.64
Scapular abduction	13	28.89	11	24.44	73.33	0.32
Scapular winging	33	73.33	31	68.89	77.78	0.46
Scapular internal rotation/tilt	2	4.44	6	13.33	86.67	0.20
Scapular elevation	0	0	0	0	—	.^a^
Humerus anterior glide	5	11.11	10	22.22	80.00	0.30
Humerus superior glide	6	13.33	8	17.78	91.11	0.66
Humerus medial rotation	0	0	0	0	—	.^a^

*Note*: ^a^ represents the measure of association was not computed because at least one response for the item was a constant.

Since the subcategory identifications were not mutually exclusive, a patient could therefore be identified as having more than one subcategory syndrome. Overall, the scapular winging was the most frequently identified by both examiners, followed by scapular depression and scapular downward rotation. For humeral syndrome, superior gliding syndrome was the most frequently observed in patients with shoulder pain.

The intertester reliability between two examiners of each subcategory syndrome are also presented in Table 3. The percentages of agreement of the MSI subcategory syndromes ranged from 73% to 91%. The scapular depression and humeral superior gliding syndromes had substantial levels of agreement (K= 0.64 and 0.66) in patients with shoulder pain. The scapular downward rotation and winging had moderate agreement (K= 0.57 and 0.46). The scapular abduction and humeral anterior glide syndrome had fair agreement (K= 0.32 and 0.30) and scapular internal rotation/tilt had poor agreement (K= 0.20). None of the examiners identified the movement impairment of scapular elevation and humeral medial rotation syndromes in any patient with shoulder pain, therefore the kappa value was not computed.

From the results, the individual subcategory syndromes might have acceptable reliability or agreement between the two examiners, but the overall agreement and kappa value of the MSI classification of subcategory syndromes was slight (K=0.112; 95% CI 0.048–0.176). Table 4 shows the 2×2 table for each subcategory syndrome including the frequency and the percentage of agreement.

**Table 4.** The frequency and the percentage of agreement of each subcategory syndromes in patients with shoulder pain.

		Classification by Examiners 2								
Subcategory syndromes		DW	DP	AB	W	IR/T	E	AG	SG	MR	Total
Classification by Examiner 1	DW	12	9	3	11	3	0	5	2	0	45
		(26.67%)	(20.00%)	(6.67%)	(24.44%)	(6.67%)	(0%)	(11.11%)	(4.44%)	(0%)	(16.79%)
	DP	10	21	7	16	6	0	7	5	0	72
		(13.89%)	(29.17%)	(9.72%)	(22.22%)	(8.33%)	(0%)	(9.72%)	(6.94%)	(0%)	(26.87%)
	AB	3	7	6	7	1	0	3	4	0	31
		(9.68%)	(22.58%)	(19.35%)	(22.58%)	(3.23%)	(0%)	(9.68%)	(12.90%)	(0%)	(11.57%)
	W	14	14	6	27	7	0	8	5	0	81
		(17.28%)	(17.28%)	(7.41%)	(33.33%)	(8.64%)	(0%)	(9.88%)	(6.17%)	(0%)	(30.22%)
	IR/T	0	1	1	2	1	0	0	0	0	5
		(0%)	(20.00%)	(20.00%)	(40.00%)	(20.00%)	(0%)	(0%)	(0%)	(0%)	(1.87%)
	E	0	0	0	0	0	0	0	0	0	0
		(0%)	(0%)	(0%)	(0%)	(0%)	(0%)	(0%)	(0%)	(0%)	(0%)
	AG	4	2	1	5	3	0	3	0	0	18
		(22.22%)	(11.11%)	(5.56%)	(27.78%)	(16.67%)	(0%)	(16.67%)	(0%)	(0%)	(6.72%)
	SG	1	2	2	4	0	0	2	5	0	16
		(6.25%)	(12.50%)	(12.50%)	(25.00%)	(0%)	(0%)	(12.50%)	(31.25%)	(0%)	(5.97%)
	MR	0	0	0	0	0	0	0	0	0	0
		(0%)	(0%)	(0%)	(0%)	(0%)	(0%)	(0%)	(0%)	(0%)	(0%)
	Total	44	56	26	72	21	0	28	21	0	268
		(16.42%)	(20.90%)	(9.70%)	(26.87%)	(7.84%)	(0%)	(10.45%)	(7.84%)	(0%)	(100%)

*Note*: DW = downward rotation, DP = depression, AB = abduction, W = wing, IR/T = internal rotation/anterior tilt, E = elevation, AG = anterior glide, SG = superior glide, MR = medial rotation.

## Discussion

This study examined the intertester reliability of the MSI classification in patients with shoulder pain. The percent agreement to identify syndromes between two physical therapists who had different levels of experience was 71.11% with fair level of agreement (K=0.34). The percent agreement range of scapular subcategories syndrome identification was 73–87% and humeral subcategories syndrome was 80–91%. The identification of subcategories syndrome also had agreements levels ranged from poor to substantial i.e., kappa from 0.20 to 0.66. Substantial level of agreements was observed in the diagnosis of scapular depression and the humeral superior gliding syndromes.

The varied levels of agreement in this study was possibly due to two main factors i.e., symptom fluctuation between two examination sessions, and the different evaluation skills of two examiners. First, this study assessed the intertester reliability by having two assessors independently examined the patients. The advantage of blinding the assessors from each other is that it would better reflect the nature of clinical practice. However, two separate examination sessions might bring about different responses of patients especially in symptom aggravation and relieving during the primary and secondary tests which was the key issue for determining subcategory syndromes. For this, we monitored the pain intensity before and after each examining session. The differences of pain levels at the beginning of two sessions were not more than 10 mm. However, some previous studies have raised concerns that repeated examination of a patient is likely to change the patient’s presentation and adversely impact the assessment of reliability.^12,18,19^

Compared with a previous study^15^ that investigated the intertester reliability of the MSI classification among three novice physical therapists in patients with knee pain, our study had slightly lower level of agreements. In the knee pain study, only one examiner performed the MSI evaluation and the other two examiners observed and assigned the diagnosis to avoid the effect of repeated testing on each patient. However, the cues from examination responses might lead to better agreements among examiners. Moreover, another MSI reliability study in patients with low back pain^11^ found almost perfect agreement when having the examiners classified the patients into movement impairment subgroups syndrome using the same recorded data.^10^ In fact, the paper case method also removed other confounding factors such as examination performance which highly influenced on the reliability.

Second, the different clinical experiences, both general clinical practice and the MSI concept, of two examiners in this study might be important factors affecting level of agreements. The differences of experience in musculoskeletal management might influence the agreements of examination because the MSI evaluation composed of the observation and manual skills commonly used in the physical therapy clinic. Similar to our study, Harris-Hayes and Van Dillen^10^ assessed the intertester reliability of the MSI classification of low back pain which two examiners using separate examination sessions with standardized physical examination form. Compared to our results, their results were more reliable (83% agreement, K– 0.75–0.99). However, both examiners in their study had over 10 years of clinical experience and one examiner was a certified clinical specialist in orthopedic field and used the MSI concepts in her clinical practice for seven years while another examiner was new for the MSI approach.

The intensive training session and the reference manual were used to standardize the examination and decision procedure since these processes were suggested to be effective to improve the level of agreements of the movement diagnosis.^10,15^ Harris-Hayes and Van Dillen^10^ suggested that the specific guideline for each test item in details and explicit rules of classification, as well as rigorous training were the key for improving the level of reliability. In addition, the strict practice and training of examiners might reinforce the confidence of clinical judgments.^10^ With the intensive training session, the percent agreements in our study were generally acceptable for both subgroup and subcategory syndromes identifications. Although both examiners did not learn from the developer of the concept, they could apply the MSI approach to evaluate and identify the MSI subgroup and subcategory syndromes with somewhat acceptable agreement in the patients with shoulder pain. This would make greater generalizability of the use of this concept among physical therapists.

Another concern of the results was the use of kappa statistics which has the chance correction reliability coefficient. The low kappa coefficient in some categories seemed to relate to the skewed response distribution i.e., the small number of some response categories due to the characteristics of the study sample.^20^ For examples, the numbers of responses in “humeral syndrome only” and the subcategories of “scapular internal rotation/tilt” were very low and these were corresponded with the low kappa coefficients of these two categories. There were also two subcategories i.e., scapular elevation and humeral medial rotation which were not identified in any participants in this study and the kappa value could not be calculated. More studies which used greater variety of patients with symptoms and examination responses are then required to confirm the agreement of therapists.

Additionally, the great number of subcategory syndromes might contribute to the poor overall agreement of the MSI classification of shoulder subcategory syndromes. Shoulder classification has nine subcategory syndromes compared with five subcategory syndromes for low back pain^10-12^ and six subcategory syndromes for knee disorders.^13-15^ With greater number of subcategory syndromes and inadequate number of subjects presented in each subcategory syndrome, the computed kappa value would be low. Moreover, the reliability is the prerequisite for validity of a classification system. The low reliability of the MSI classification for shoulder disorders will then threaten its validity.

Another concern which might distress the validity of a shoulder MSI classification system examined in this study is the non-mutually exclusive of its subcategories. The subcategories of this classification might need to be reviewed to meet the fundamental requirement for a valid classification system i.e., mutually exclusive and exhaustive category.^21^

The clinical assessment of humeral and scapular position and motion are apparently challenging due to the large muscles mass and complex movement patterns.^22^ The reported kappa coefficients for identifying scapular abnormal positions and movements in previous studies were commonly poor to moderate.^23-26^ The results of kinematic studies of humeral movement impairments were also inconsistent.^27,28^

The results suggested that the movement and alignment alteration of both scapula and humerus were coexisting with shoulder pain. The scapular syndromes were identified in 96–100% of participants in this study. The patterns most observed were winging (70–72%), depression (48–56%), and downward rotation (34–38%). A previous study^29^ suggested that the scapular kinematic changes were observed in 68–100% of individuals with shoulder injury. For shoulder dysfunction, the altered scapular movements were reported to be decreased posterior tilting and upward rotation, and increased scapular elevation.^30^ Specifically, the most frequent findings for SIS were reduced scapular posterior tilting, reduced upward rotation, increased internal rotation, as well as increased clavicular elevation.^31^ Additionally, increased humeral head superior or anterior translation had been found in subjects with impingement.^32,33^ However, different alterations of scapular movement were reported in different shoulder pathologies.^31^

There were some limitations in this study. First, the symptom status of patients over two examination sessions might change, although we monitored subjective symptom by rating the pain intensity at the beginning and end of each session and made certain that the symptom levels were equal. Previous investigators have suggested that poor reliability for items related to the symptoms elicited may have resulted from using a repeated testing (test–retest) design.^10,11,15^ The effect of the repeated assessment and corrected alignment and movement might also cause learning and changing of the pattern of movement and symptom response. Another limitation was that most subjects in this study had mild to moderate levels of shoulder pain and disability. The results might be different if subjects with greater pain and disability levels were included. There were also less number of subjects with some movement impairment patterns including humeral syndrome only and scapular internal rotation/tilt. None of subjects was identified as having scapular elevation and humeral medial rotation. The examiners in this study had different clinical experiences and education regarding the MSI approach. Both of them also did not receive the training from the approach developer. More research is needed to concentrate on examiners with wider ranges of characteristics in terms of clinical experiences and familiarity to the classification system.

## Conclusion

This study showed generally acceptable to poor reliability of two physical therapists with different levels of experience to classify the MSI. However, the novice therapist with intensive training was able to diagnose patients with shoulder pain with fair agreements referenced with the more experienced therapist. The great number of categories of the shoulder MSI classification system might be a factor for the poor agreement level which considering the possibility of the agreement occurring by chance. The insufficient agreements in this study were also possibly associated to symptom changes between two separate examination sessions, and the absence of patients classified in some subcategories.

## Acknowledgments

The authors would like to thank all participants and personnel of all data collection settings. The doctoral education of first author was supported financially by Siriraj Development Foundation, Faculty of Medicine, Siriraj Hospital, Mahidol University.

## Conflict of Interest

The authors have no conflict of interest relevant to this paper.

## Funding/Support

This research did not receive any grant from funding agencies in the public, commercial, or not-for-profit sectors.

## Author Contributions

All authors conducted the conception and design of the study. VM and TP performed the assessment procedure, an acquisition, analysis, interpretation of the data, and drafting of this paper. All authors revised and approved the final paper.

## Appendix A. Classification of Movement System Impairment of Shoulder Girdle

**Table A.1.** 

Subcategory syndromes	Alignment impairments	Movement impairments
1. Scapular syndrome		
1.1. Scapular downward rotation	– The vertebral border of scapula is not parallel to midline and the inferior angle is closer to midline compared with the superior border or root of scapular spine. – The scapula may be adducted in resting position. – Forward shoulder. – Increased slope of shoulder level. – Abduction of the humerus can be secondary to the downward rotated position of the scapula.	– Insufficiency of scapular upward rotation or glenohumeral elevation or both during the final phase of shoulder elevation. – The scapular downward rotation during the first 60^∘^ of shoulder flexion and 30^∘^ of shoulder abduction. – The inferior angle of the scapula not reach to the midaxillary line of the thorax during shoulder full elevation.
1.2. Scapular depression	– The superior angle of scapula is lower than the second of thoracic vertebral spinous process. – The clavicles is placed on horizontal or slightly lower lateral than medial. – Slope of shoulder is increased. – Involved arm is longer than uninvolved arm.	– Insufficiency of scapular elevation during shoulder flexion and abduction. – The acromion process depressed in the first 90^∘^ of arm elevation or not elevated after 30^∘^ of arm elevation.
1.3. Scapular abduction	– The distance between the vertebral of spinous process and vertebral border of scapula is greater than three inches and resting scapular greater than 30^∘^ and anterior to frontal plane. – The position of glenohumeral joint is placed on anterior than normal alignment.	– Excessive scapular abduction during shoulder flexion and abduction. – Axillary border of scapula protrudes to lateral greater than 1/2 inches beyond the thorax at the end of shoulder flexion and abduction. – In prone position, scapular abduct during shoulder lateral rotation. – Scapulohumeral rhythm altered to 1:1 ratio during the phase of shoulder flexion from about 90–180^∘^.
1.4. Scapular winging	– The medial border of scapular is prominent from rib cage and scapular internal rotation is more than 40^∘^.	– The vertebral border of scapular winged during shoulder flexion and abduction as well as during return to shoulder flexion.
1.5. Scapular internal rotation and anterior tilt	– For scapular internal rotation, the scapula is rotated more than 30–40^∘^ anterior to frontal plane. – For scapular tilting, the scapular is tipped forward from rib cage and prominence of inferior angle of scapula and the scapular anterior tilt more than 15^∘^.	– Insufficiency of scapular external rotation and posterior tilt at the end range of arm elevation.
1.6. Scapular elevation	– The alignment of scapula is above 2nd and 7th thoracic vertebral spinous process. – Decreased slope of shoulder girdle and increased upward slope of clavicle.	– Excessive of scapular elevation at any period of shoulder elevation.
2. Humeral syndrome		
2.1. Humeral anterior glide	– Greater than one third of humeral head is positioned anterior to acromion process. – Shoulder is in forward position. – The humeral head is anterior to the distal end of humerus. – The indentation is observed below the acromion in the posterior aspect.	– Excessive or abnormal of humeral anterior gliding during shoulder abduction, horizontal abduction, return to flexion, medial or lateral rotation, and elbow extension. – Humeral anterior gliding might occur during prone position and active shoulder lateral rotation than passive. – Humeral anterior gliding and pain might occur during shoulder rotation in the frontal plane than scapular plane. – Horizontal adduction might produce pain at anterior shoulder due to insufficiency of humeral posterior gliding. – Accessory joint motion increased anteriorly and decreased posteriorly.
2.2. Humeral superior glide	– Decreased subacromial space. – The humerus is in abduction position relative to scapula. – The scapula is positioned as depression or downward rotation.	– Insufficient inferior gliding of humerus head during shoulder elevation. – Excessive humeral superior glide during shoulder flexion, abduction, and medial or lateral rotation. – Decreased distance between humeral head and base of neck at the end range of arm elevation.
2.3. Humeral medial rotation	– Medial rotation of humerus in resting position. – Forward shoulder.	– Insufficiency of lateral rotation of humerus during shoulder elevation. – Excessive humeral medial rotation during shoulder flexion and abduction.
